# Cost Evaluation of a Government-Conducted Oral Cholera Vaccination Campaign—Haiti, 2013

**DOI:** 10.4269/ajtmh.16-1023

**Published:** 2017-10-18

**Authors:** Janell A. Routh, Nandini Sreenivasan, Bishwa B. Adhikari, Lesly L. Andrecy, Margarette Bernateau, Taiwo Abimbola, Joseph Njau, Ernsley Jackson, Stanley Juin, Jeannot Francois, Rania A. Tohme, Martin I. Meltzer, Mark A. Katz, Eric D. Mintz

**Affiliations:** 1 Division of Viral Diseases, National Center for Immunization and Respiratory Diseases, Atlanta, Georgia;; 2 Division of Preparedness and Emerging Infections, National Center for Emerging Infectious Diseases, Centers for Disease Control and Prevention, Atlanta, Georgia;; 3 Field Epidemiology Training Program, Direction d’Epidémiologie de Laboratoire et de Recherches (DELR), Ministère de la Santé Publique et de la Population (MSPP), Port-au-Prince, Haiti;; 4 Global Immunizations Division, Center for Global Health, Centers for Disease Control and Prevention, Atlanta, Georgia;; 5 Immunization Program, UNICEF, Petion-ville, Haiti;; 6 US Centers for Disease Control and Prevention, US Embassy, Port-au-Prince, Haiti;; 7 Directeur du Programme Elargi de Vaccination (DPEV), MSPP, Ave Maïs Gaté, Port-au-Prince, Haiti

## Abstract

The devastating 2010 cholera epidemic in Haiti prompted the government to introduce oral cholera vaccine (OCV) in two high-risk areas of Haiti. We evaluated the direct costs associated with the government’s first vaccine campaign implemented in August–September 2013. We analyzed data for major cost categories and assessed the efficiency of available campaign resources to vaccinate the target population. For a target population of 107,906 persons, campaign costs totaled $624,000 and 215,295 OCV doses were dispensed. The total vaccine and operational cost was $2.90 per dose; vaccine alone cost $1.85 per dose, vaccine delivery and administration $0.70 per dose, and vaccine storage and transport $0.35 per dose. Resources were greater than needed—our analyses suggested that approximately 2.5–6 times as many persons could have been vaccinated during this campaign without increasing the resources allocated for vaccine delivery and administration. These results can inform future OCV campaigns in Haiti.

## INTRODUCTION

After the January 2010 earthquake, Haiti experienced one of the largest cholera epidemics ever recorded in the Western Hemisphere. As of November 30, 2016, over 800,000 cases and 9,500 deaths had been reported to the National Cholera Surveillance System.^1^ In February 2013, the Haitian Ministry of Health and Population [French acronym: MSPP] launched a cholera elimination plan.^2^ While the plan focused on long-term solutions addressing water, sanitation, and hygiene (WASH) infrastructure, MSPP proposed oral cholera vaccine (OCV) campaigns as an additional approach to control the spread of cholera. OCVs have been studied in situations for endemic prevention and epidemic response, and found to be effective. Of the two vaccines licensed at the time of the OCV campaign in Haiti, Dukoral and Shanchol, Shanchol, a bivalent, killed, whole cell OCV which is given in two doses separated by at least 14 days, was used for its ease of administration and lower cost.^3^ The effectiveness of Shanchol was 86% at 6 months during a cholera outbreak in Guinea; this effectiveness has been consistent across other evaluations.^4,5^ Five-year data shows continued effectiveness of two doses to be 65%,^6^ and recently an evaluation of a single-dose regimen showed a protective efficacy of 40% against all episodes of cholera and 63% against severely dehydrating cholera in an urban area of high endemicity.^7^

From August 5–9, August 26–30 to September 9 and 10, 2013, MSPP conducted its first OCV campaign. Whereas two previous OCV campaigns had been conducted by nongovernmental organizations in Haiti, this was the first government-operated OCV campaign.^8,9^ This was also one of the few campaigns in Haiti to target an adult population. To guide future decisions and planning for OCV campaigns, we conducted an evaluation of the major programmatic costs of this campaign. Furthermore, we assessed the efficiency of campaign resource use to identify areas for potential improvement in subsequent campaigns.

## METHODS

### Site description.

**Figure 1.**
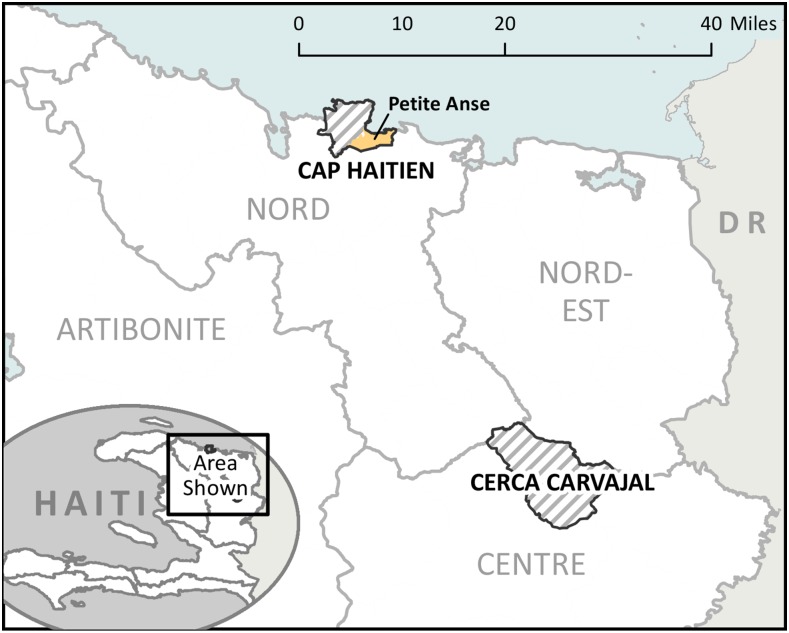
Geographic location of the vaccination campaign sites of Cerca Carvajal and Petite Anse, Haiti, 2013. This figure appears in color at www.ajtmh.org.

For the OCV campaign, MSPP chose two sites that were at increased risk for cholera because of limited safe water sources and sanitation facilities, and poor access to health care: Cerca Carvajal, a rural, mountainous commune of approximately 20,917 vaccine-eligible people in Center Department; and the communal section of Petite Anse, in the coastal city of Cap Haitien in the North Department, with a vaccine-eligible population of 86,989 (total target population, 107,906) (Figure 1). These two areas had demonstrated persistently high attack rates for cholera (10.1–37.0%) since 2010.^10^ Vaccine eligibility was defined as any person ≥ 1 year of age, living in the catchment area of Cerca Carvajal or Petite Anse, and not known to be pregnant during the campaign dates.

### Campaign organization.

Campaign planning began in April 2013. A planning committee was formed that included members from MSPP, UNICEF, The Pan American Health Organization (PAHO), the US Centers for Disease Control and Prevention (CDC), Partners in Health and GHESKIO (The Haitian Group for the Study of Kaposi’s Sarcoma and Opportunistic Infections). UNICEF donated 238,700 doses of Shanchol for the campaign, shipped them to Haiti in April, and stored them in the government’s central vaccine warehouse near the Port-au-Prince airport for 4 months until the beginning of the campaign. During August 5–9 and August 26–30, two rounds of the vaccination campaign were held in Cerca Carvajal and Petite Anse. However, the second round in Petite Anse had to stop on August 28 because an underestimation of the targeted population led to a stock-out of vaccines. A second batch of 29,925 doses of vaccine was donated by Médecins Sans Frontières (MSF) in South Sudan to cover this deficit. The vaccine arrived in Haiti on September 5 and allowed completion of the vaccination campaign in Petite Anse on September 9 and 10, 2013.

The branch of MSPP that is responsible for vaccination activities, the Direction du Program Elargi de Vaccination (DPEV), created vaccine delivery teams consisting of three staff (crieur [announcer], enregistreur [record-keeper] and vaccinateur [vaccinator]) and hired supervisors to monitor approximately 3–5 teams each. They recruited staff from a pool of trained vaccinators and social mobilizers that had worked on routine immunization services and other vaccine campaigns throughout Haiti. MSPP conducted a 2-day training for all supervisors and team members the week before the first round of the campaign and a 2-hour refresher training before the second round. Demand for OCV was stimulated by mobilizers active in the communities during the weekend before the first dose was offered. MSPP distributed information about the vaccination campaign to a very limited area before the campaign started to minimize the possibility that large crowds of people from outside the vaccination area would come to get vaccinated. Megaphones, posters, flyers, and green tee-shirts worn by members of the campaign advertised the purpose and logistical aspects of the campaign to people within the two vaccination areas. Each vaccination team performed the following activities on vaccination days: screening for eligibility, obtaining verbal consent from each participant, administering vaccine, filling tally sheets, monitoring for immediate adverse events, completing and distributing vaccination cards, collecting used vaccine vials and aluminum and rubber lids at the end of each session, and returning waste to the designated post.

Each person who received a complete first dose of OCV was given a vaccine card specifically designed for the OCV campaign. The card included information about age and sex of the vaccinated person, their name, and the date and place the first dose was administered. Vaccinees were told to keep their vaccination cards and to bring them back for the second dose. During the second round, MSPP campaign staff checked for proof of receipt of the first dose, added the date of receipt of the second dose, and recorded the name, age, sex, address, and phone number of vaccine recipients in vaccination registration books. No registers were available for the first round.

Given the geographical differences between rural Cerca Carvajal and urban Petite Anse, MSPP tailored each campaign to the geography to maximize vaccine delivery. In the rural location of Cerca Carvajal, 42 vaccination teams operated daily for the full 10 days of the campaign. Teams administered vaccine at fixed sites such as health centers and schools, which were more practical for this mountainous area of the country. In Petite Anse, 174 vaccination teams administered vaccine on street corners, designated neighborhood locations, and at 10 fixed post sites in schools, churches and health centers. On the last day of each round of the campaign, teams canvassed neighborhoods in Petite Anse going door to door to look for unvaccinated persons.

### Cost analysis.

#### Campaign costs estimation.

We estimated OCV campaign costs from the vaccine providers’ perspective. To provide a complete set of costs, we included the expenditures by MSPP for campaign administration and the costs of the vaccine, even though the vaccine was donated. Costs not borne by MSPP, such as work productivity losses and transport costs for people receiving the vaccine, were excluded from the analysis.

We evaluated costs incurred by both precampaign activities (e.g., training, vaccine storage) and the campaign itself during the period August–September 2013. We collected data about campaign-related expenditures by analyzing budgets submitted after the campaign ended by the two departments where the campaign occurred. We validated projected expenditures in the two budgets by comparing them to expenditure invoices. We collected campaign-related receipts and records maintained at departmental offices for each vaccination area and compared them to the original budgeted input items to estimate total costs. For items that lacked expenditure invoices, we interviewed key management and finance staff involved in the campaign to best estimate cost.

Costs were divided into the following categories:

- Vaccine costs, which included the market value of the vaccine, its shipment, and storage (cost of the international shipment of the vaccine, customs clearance, and warehouse storage costs);
- Site-specific costs, including items purchased and paid for at the two vaccination sites (local storage and distribution costs; cold-chain maintenance and logistic support; social mobilization activities; staff training; incentives and travel support for vaccinators, supervisors and cold-chain handlers; other transport costs; office supplies; field supplies [vaccine carriers, ice packs, waste bags]); and waste management;
- Central-level costs, including items purchased and paid for in Port-au-Prince and delivered to the field sites (per diem for supervisors, drivers and logisticians from Port-au-Prince, phone credit, vehicle fuel, lease and maintenance costs, and centrally purchased supplies [vaccine cards, vaccine registers, and campaign promotion media and materials including OCV campaign tee-shirts and ball caps, campaign banners, pamphlets and posters]).

Central-level costs were weighted based on the number of vaccine doses administered and split between the two campaign areas. The central level and site-specific costs both pertain to campaign operations and are also referred to as “vaccine delivery” costs in the results and Table 1. For members of the vaccine team who worked a regular job, we considered the per diem they received to be campaign-related, but we did not consider their usual salary to be a campaign-related cost. We did not consider staff time spent on program planning, research, and evaluation activities, and costs of existing infrastructure (such as refrigerators used to store the vaccine, office space, and utilities) to be campaign-related costs. Campaign-related costs included only additional resources, such as promotional materials and vaccinators, which were necessary to ensure vaccine delivery during the campaign.

**Table 1:** Major cost categories and expenses from the oral cholera vaccine campaign, Haiti, 2013

	Cerca Carvajal (rural)		Petite Anse (urban)		Overall	
Cost item	Total costs ($)	% of total	Total costs ($)	% of total	Total costs ($)	% of total
Vaccine costs*	96,269	69.3	376,597	77.6	472,866	75.8
Site-specific costs						
Local personnel	23,174	16.7	63,687	13.1	86,861	13.9
Field and office supplies	3,103	2.2	2,276	0.5	5,379	0.9
Local vaccine management†	4,864	3.5	4,614	1.0	9,478	1.5
Social mobilization	1,919	1.4	4,181	0.9	6,100	1.0
Central level costs‡	9,636	6.9	33,206	6.9	42,842	6.9
Total costs	138,966	100	484,561	100	623,527	100
	Cost per dose	% of total	Cost per dose	% of total	Cost per dose	% of total
Vaccine cost per dose	1.85	58	1.85	66	1.85	64
Vaccine shipping and storage	0.35	11	0.35	12	0.35	12
Vaccine delivery	0.98	31	0.62	22	0.70	24
Total cost per dose administered	3.18	100	2.82	100	2.90	100

* Doses were donated to the government of Haiti but priced here at the market value of $ 1.85/dose. See text for further details. Includes vaccine shipping and storage costs.

† Local storage and distribution costs, cold-chain maintenance and logistical support.

‡ Per diem for staff from Port-au-Prince, phone credit, vehicle fuel/lease/maintenance, supplies, and campaign promotional media and materials.

We entered cost data into SurvCost, a spreadsheet tool developed by the US CDC to track costs of programmatic activities using budget and expense information.^11^ We compiled and analyzed campaign-related costs for each of the two vaccination areas and calculated the approximate cost per dose of vaccine administered. Vaccine tally sheets and registers kept by campaign supervisors and data managers at each site recorded the number of OCV doses dispensed and persons vaccinated. We calculated costs in 2013 prices in US dollars ($) based on an exchange rate of $1–43 Haitian Gourdes at the time of the campaign. We priced the vaccine at $1.85 per dose, the market price of Shanchol at the time of the campaign for public health programs in low income and middle income countries.^12^

#### Campaign vaccine delivery capacity.

We collected data related to vaccine delivery capacity, defined as the number of doses that could be delivered per vaccine team per day given no resource constraints, such as transportation issues or vaccine stock-outs. Two evaluation teams, composed of epidemiologists from CDC and students with CDCs Field Epidemiology Training Program in Haiti, collected data related to vaccine delivery capacity during the days of campaign operation. One team visited the central post in Cerca Carvajal and the second team visited each of ten fixed vaccine posts in Petite Anse to record the number of vaccine teams in the field, the number of vaccine doses delivered, the number of hours worked by each team, and the vaccine supplies used by each vaccine team.

The evaluation teams did not observe each vaccine team every day in either location because of the large number of teams and the distances between them. In Cerca Carvajal, the evaluation team observed one or two field teams for the first 3 days of each round. In Petite Anse, the evaluation team went to each of the 10 fixed post sites daily and observed two to three vaccine teams at each site for a minimum of 30 minutes. We used a timer to record the number of seconds it took to vaccinate a person, prepare the vaccination card, and record the personal demographic information into the vaccine register. We estimated the average number of administered doses per day for each 5-day vaccination period because we did not have resources to adequately audit the accuracy of daily vaccine delivery logs. We then divided these averages by the number of teams vaccinating each day and their hours worked to provide an estimate of doses administered per team per day, our measure of vaccine delivery capacity.

We then calculated the range of daily vaccine delivery capacity (defined in vaccine doses delivered [*D*]) by dividing a plausible range of vaccination hours available (*T*) (i.e., allowing for travel time, time for rest, etc.) by the observed time needed to administer and record a single vaccine dose (*A*_vd_) to get the number of doses of vaccine that could be delivered (*D*) in that time range (*D* = *T*/*A*_vd_) per vaccine team. When estimating capacity, we assumed that there would be both sufficient vaccine doses available and persons needing vaccination, such that teams would be continuously occupied during the time available. The purpose of this exercise was to provide a basis for optimizing the delivery of vaccine doses for subsequent campaigns. This information could be considered for future microplanning related to OCV vaccination campaigns in Haiti.

#### Ethical considerations.

The Haiti Ethics Board approved the study and the Human Subjects Protection Office at CDC determined this project to be a programmatic evaluation of a proven public health practice.

## RESULTS

### Cost.

The total cost of the campaign was $623,528. Administrative records indicated that the campaign dispensed 215,295 OCV doses; 113,045 during the first round and 102,250 during the second round. Two-dose coverage estimates for the campaign were 76.8% in Cerca Carvajal and 62.5% in Petite Anse.^13^

Vaccine delivery costs totaled $150,660, or 24% of the overall costs of the campaign. The cost of vaccine doses, shipping to Haiti, and cold storage accounted for 76% of the total cost. The major expenses of the campaign were the cost of vaccine doses (64%) and personnel (13.9%) (Table 1). Based on 215,295 doses administered, the overall cost per dose of vaccine administered was $2.90. The largest component of this cost was the cost of the vaccine itself at $1.85 per dose. Overall $0.35 per dose was spent on vaccine storage and shipping to Haiti, and $0.70 per dose was spent on vaccine delivery costs. Vaccine wastage, due to spilled doses, broken vials, or refusals, was negligible in each location—0.04% in Cerca Carvajal and 0.02% in Petite Anse—and therefore not included in the overall calculations. In total, vaccine teams delivered 43,768 OCV doses in Cerca Carvajal and 171,527 doses in Petite Anse. Despite dispensing almost four times as many doses in Petite Anse, vaccine delivery costs were higher in Cerca Carvajal (31% versus 22%), reflecting the rural nature of this location and difficulty transporting vaccine to target destinations.

### Capacity.

Including vaccine administration and vaccine card provision, we observed an average vaccination time during the first round of the campaign of 35 seconds per person in both Cerca Carvajal and Petite Anse. Vaccine administration time was similar for administration to a child or adult. During the second round of the campaign when MSPP added a vaccine register to record demographic information of persons vaccinated, vaccination time averaged 60 seconds per person, whether an adult or child.

Work activities for the 8-hour work day were similar between the two sites. In Cerca Carvajal, vaccine teams spent 4–5 hours delivering vaccine. The teams spent 1 to 2 hours traveling to remote vaccination posts and the remaining time was spent for lunch, tracking inventory and record keeping. In Petite Anse, vaccine teams spent 5 to 6 hours delivering vaccine. Travel time was not included in the work day, as the workers presented to their vaccine post straight from home. The teams spent the remaining 2 to 3 hours resting between vaccine administration, on lunch break, tracking inventory, and record keeping (Table 2).

**Table 2:** Capacity variables for the oral cholera vaccine campaign, Haiti, 2013

	Cerca Carvajal (rural)		Petite Anse (urban)	
Vaccine campaign round	1	2	1	2
Number of teams	42	42	174	174
Persons per team	3	3	3	3
Number of hours spent vaccinating persons per team per day	4–5	4–5	5–6	5–6
Total number of doses administered	21,894	21,874	91,151	80,376
Time per dose administered	35 seconds	60 seconds	35 seconds	60 seconds

Using these work hour approximations, we calculated the vaccine delivery capacity (*D* = *T*/*A*_vd_) per team to be between 411 and 514 vaccine doses delivered per day (4 hours [14,400 seconds]/35 seconds per vaccine dose delivery = 411 and 5 hours [18,000 seconds]/35 seconds per vaccine dose delivery = 514) in the rural setting of Cerca Carvajal, and between 514 and 617 vaccine doses delivered per team per day (5 hours [18,000 seconds]/35 seconds per vaccine dose delivery = 514 and 6 hours [21,600 seconds]/35 seconds per vaccine dose delivery = 617) in the urban setting of Petite Anse. After adding the time for names to be recorded in the vaccine register, which increased the total vaccine dose delivery time to 60 seconds/dose, the capacity was 240–300 vaccine doses delivered per team per day in Cerca Carvajal, and 300–360 doses delivered per team per day in Petite Anse.

To calculate the average number of doses actually delivered per team per day during the campaign, we assumed that the total doses for each site and each round were distributed equally over 5 days. We calculated the average number of doses delivered in Cerca Carvajal to be 104 vaccine doses per team per day during both rounds 1 and 2 (21,894 doses/42 teams/5 days = 104), and in Petite Anse to be 105 doses per team per day (91,151 doses/174 teams/5 days = 105) during round 1, and 92 (80,376 doses/174 teams/5 days = 92) vaccine doses per team per day during round 2 (Table 3). Although we could not adequately track daily vaccine usage, and therefore, used a 5-day average, as previously mentioned, evaluation teams were able to account for 91–99% of daily OCV usage totals in both sites. Based on approximate daily totals, we calculated daily doses delivered per team (Supplemental Appendix).

**Table 3:** Observed and theoretical oral cholera vaccine campaign capacity, Haiti, 2013

	Cerca Carvajal (rural)				Petite Anse (urban)			
Vaccine campaign round	1		2		1		2	
Average number of doses delivered per day*	104		104		105		92	
Range of daily vaccination capacity†	Min	Max	Min	Max	Min	Max	Min	Max
Theoretical capacity‡	411	514	240	300	514	617	300	360
Actual as a % of theoretical	20–25%		35–43%		17–20%		26–31%	

* Calculated assuming equal number of vaccine doses delivered per day.

† Capacity range based on 4–5 hour vaccination time in the rural area and 5–6 hour vaccination time in the urban area.

‡ Theoretical capacity = expected number of vaccine doses delivered for the resources allocated per vaccine team per day.

The average number of doses delivered per day during the campaign was considerably less than the calculated vaccine delivery capacity (104 versus 411–514 for Cerca Carvajal in round 1 and 104 versus 240–300 in round 2; 105 versus 514–617 for Petite Anse in round 1 and 92 versus 300–360 in round 2). These findings suggest that approximately 2.5–6 times as many persons could be vaccinated without increasing campaign resources if the doses of vaccine were available as a constant supply, and persons receiving the vaccines did not decrease over time, which were inefficiencies that affected this particular campaign (Table 3).

## DISCUSSION

We estimated the cost of the OCV campaign to be $623,528—nearly $3/dose. Vaccine alone accounted for almost two-thirds of the total costs. Although the vaccine in this campaign was a donation from UNICEF, future vaccine campaigns must take this expense into account. The increased cost per dose in rural Cerca Carvajal ($3.18 versus $2.82) reflected the increased vaccine delivery costs in a geographically isolated area with a widely dispersed population. In Cerca Carvajal, campaign organizers augmented personnel, supplies, and transportation relative to the number of OCV doses dispensed to meet these challenges. Planning for future campaigns will need to take into account Haiti’s diverse landscape.

The per-dose cost for OCV in Haiti was relatively similar to per-dose costs reported from OCV campaigns in other countries. The cost per fully immunized person of a two-dose OCV campaign in a refugee camp in Uganda ranged from $0.53 to $3.69 excluding the cost of vaccine (Dukoral).^14^ A cost analysis in Zanzibar using Dukoral found the cost of vaccine purchase was 68% of the total cost, compared with 64% for this campaign; a similar overall proportion despite the per dose cost of OCV for the Zanzibar campaign was more expensive at $5. In the Zanzibar analysis, vaccine transport and storage costs for a much smaller amount of vaccine (51,000 doses) amounted to 4%, compared with our finding of 12% for roughly 270,000 doses.^15^ In Mozambique, the cost of a 2-dose OCV campaign with Dukoral in an urban setting amounted to $2.09 per fully vaccinated person, excluding the cost of vaccine.^16^ This is similar to our calculated cost of $1.05 per dose (or $2.10 per fully vaccinated person) excluding the cost of vaccine. Two campaigns using Shanchol, in Bangladesh and India, reported costs per dose of $1.83 (using a vaccine cost of $1.00/dose) and $2.71, respectively.^17,18^ These costs are again similar to what we calculated for the Haitian campaign. It is worth noting that each vaccine campaign is unique to a particular setting with different methods of vaccine delivery and record keeping, and different criteria for calculating costs, so that comparisons across campaigns are approximations.

After calculating the capacity of this campaign with its given resources, and estimating an expected capacity with those same resources, it is clear that this campaign operated far below capacity. In the urban area, almost 3.5–6 times as many persons could be vaccinated with the same resources, if availability of vaccine doses and if vaccine-eligible people were optimized for each team during the full work day. In rural areas, the same resources would have vaccinated 2.5–5 times as many persons. The theoretical capacity of the urban area exceeded the rural because of less time spent traveling to vaccination posts and more time available to administer OCV; however, with respect to the actual number of doses delivered, the rural site outperformed the urban. Resource allocation to the rural site likely matched the workload and vaccination needs more closely, a finding that can inform future distribution of personnel. Understanding the number of vaccinations a team can provide and its target population will help tailor the workforce to the population the campaign expects to vaccinate. Increasing efficiency by optimizing resource utilization could lower the cost of vaccination on a per dose basis. Novel methods of delivery or delivery approaches tied to other, ongoing, vaccination efforts, such as routine childhood immunization may also help decrease campaign costs. During vaccination campaigns in Haiti, routine immunization (RI) activities are deferred, leading to a drop in immunization coverage. Combining OCV campaigns with RI activities could reduce wastage of time and resources and potentially increase RI coverage.

We acknowledge several limitations to this analysis. First, it was not feasible to collect all cost data for the campaign. We did not collect information on the opportunity cost of staff time used for the campaign. We were unable to collect complete financial records for Petite Anse, and therefore, items like interdepartmental transport, locally procured office supplies, and media may be reported at less than the actual cost of these items, which may have led to an underestimation of vaccine delivery costs. Second, we did not collect the costs for preparatory planning meetings that took place from March through August. Third, we could not track persons who received both first and second doses of vaccine, and thus we were not able to calculate the cost per person fully vaccinated. Fourth, costs were estimated on an incremental basis; health system strengthening (e.g., storage facilities, vaccination locations, and additional cold-chain measures) may be needed to support larger campaigns in more remote areas. These costs were not included in this analysis. Finally, we averaged the number of vaccine doses delivered per day across the five days of each round of the campaign for our capacity calculation. In reality, the campaign delivered more vaccine doses on days 1–3 when people and vaccine were plentiful. This dropped off steeply on days 4 and 5 when there were fewer vaccine doses available and fewer people were actively seeking vaccination. For round 1 in Petite Anse, 93% of OCV doses were delivered in the first 3 days; this figure dropped to 76% for round 2. In Cerca Carvajal, 85% of doses were delivered in the first 3 days and 67% for round 2. Although the vaccine team efficiency was higher for these first three days when vaccine and people were plentiful, the daily number of doses delivered still fell below the theoretical capacity of the campaign. This suggests that resources could be reallocated to hold shorter campaigns that better match the initial public demand for services.

The costs related to a vaccine campaign are only one aspect that should guide decision-making about the introduction of a new vaccine on a national or local level. Governments and planning committees must also consider the burden and severity of the disease, the availability of other prevention and control measures, the political priority of the disease, and the effectiveness of the vaccine. The economic and social burden and the severity with which cholera moved through Haiti are well documented. Although good measures for prevention and control exist in many parts of the world, these resources are unlikely to be widespread in Haiti in the near future.

A 37-month follow-up evaluation of vaccine effectiveness in the first OCV campaign in Haiti led by GHESKIO showed continued protection at approximately 97.5%.^19^

These findings, combined with the political priority of cholera in Haiti and worldwide, make targeted vaccine campaigns in Haiti a promising tool in cholera outbreak response and in the path toward elimination.^20^

In early October, Hurricane Matthew struck the south-west portion of Haiti, causing devastation and a resurgence in cholera cases. The Haitian government, together with CDC and Partners in Health, mounted an OCV campaign in the most heavily affected areas to control the spread of the outbreak. This cost analysis will assist the government and its funding partners to predict and help reduce the expense of these ongoing campaigns.

## Supplementary Material

Supplemental Appendix.
