# Supplementary material for: Cost Evaluation of a Government-Conducted Oral Cholera Vaccination Campaign—Haiti, 2013
Source: Am J Trop Med Hyg. 2017 Oct 18;97(4 Suppl):37–42. doi: 10.4269/ajtmh.16-1023 (PMC5676633; doi:10.4269/ajtmh.16-1023)
Supplement: Supplementary file 1 [file tpmd161023.SD1.pdf]

The following are supplemental materials and will be published online only

SUPPLEMENTAL APPENDIX

Supplemental figures for the daily average doses delivered per team.

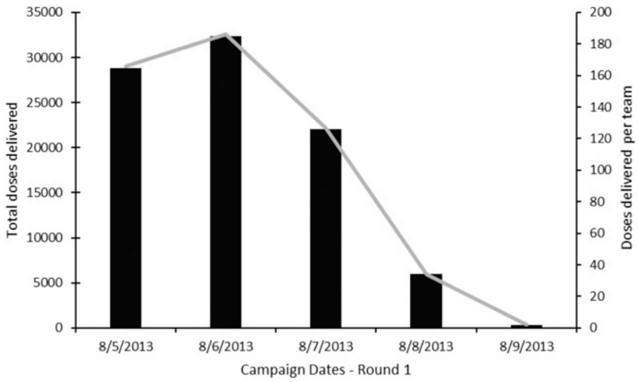

Supplemental Figure A1. Daily doses delivered—round 1 of oral cholera vaccine (OCV) campaign, Petite Anse, August 5–9, 2013.

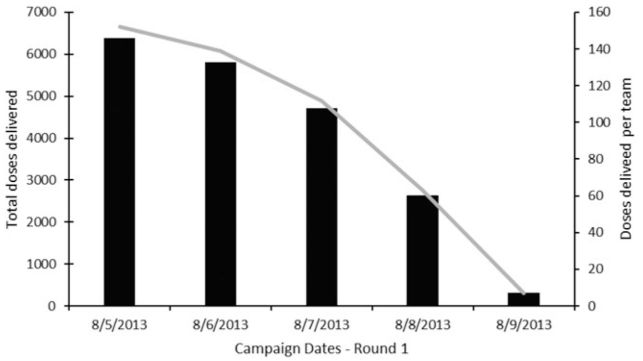

Supplemental Figure A3. Daily doses delivered—round 1 of oral cholera vaccine (OCV) campaign, Cerca Carvajal, August 5–9, 2013.

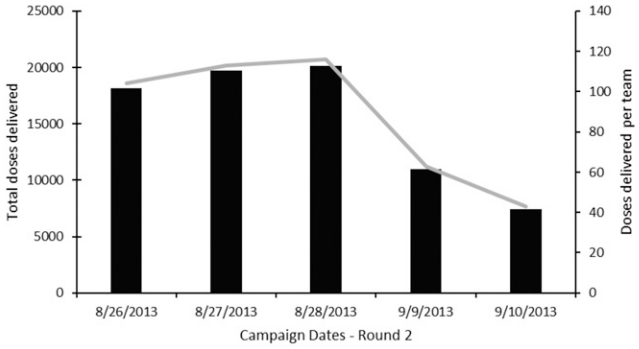

Supplemental Figure A2. Daily doses delivered—round 2 of oral cholera vaccine (OCV) campaign, Petite Anse, August 26–September 10, 2013.

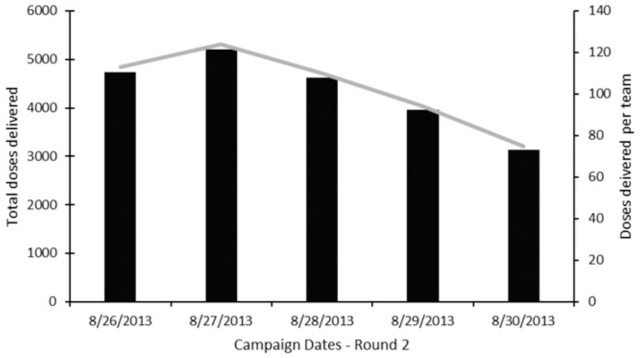

Supplemental Figure A4. Daily doses delivered—round 2 of oral cholera vaccine (OCV) campaign, Cerca Carvajal, August 26–30, 2013.
